# Yeast Modulation of Human Dendritic Cell Cytokine Secretion: An *In Vitro* Study

**DOI:** 10.1371/journal.pone.0096595

**Published:** 2014-05-09

**Authors:** Ida M. Smith, Jeffrey E. Christensen, Nils Arneborg, Lene Jespersen

**Affiliations:** 1 Health & Nutrition Division Discovery, Chr. Hansen A/S, Hørsholm, Denmark; 2 Department of Food Science, University of Copenhagen, Frederiksberg, Denmark; Institute for Virus Research, Laboratory of Infection and Prevention, Japan

## Abstract

Probiotics are live microorganisms which when administered in adequate amounts confer a health benefit on the host. The concept of individual microorganisms influencing the makeup of T cell subsets via interactions with intestinal dendritic cells (DCs) appears to constitute the foundation for immunoregulatory effects of probiotics, and several studies have reported probiotic strains resulting in reduction of intestinal inflammation through modulation of DC function. Consequent to a focus on *Saccharomyces boulardii* as the fundamental probiotic yeast, very little is known about hundreds of non-*Saccharomyces* yeasts in terms of their interaction with the human gastrointestinal immune system. The aim of the present study was to evaluate 170 yeast strains representing 75 diverse species for modulation of inflammatory cytokine secretion by human DCs *in vitro*, as compared to cytokine responses induced by a *S. boulardii* reference strain with probiotic properties documented in clinical trials. Furthermore, we investigated whether cytokine inducing interactions between yeasts and human DCs are dependent upon yeast viability or rather a product of membrane interactions regardless of yeast metabolic function. We demonstrate high diversity in yeast induced cytokine profiles and employ multivariate data analysis to reveal distinct clustering of yeasts inducing similar cytokine profiles in DCs, highlighting clear species distinction within specific yeast genera. The observed differences in induced DC cytokine profiles add to the currently very limited knowledge of the cross-talk between yeasts and human immune cells and provide a foundation for selecting yeast strains for further characterization and development toward potentially novel yeast probiotics. Additionally, we present data to support a hypothesis that the interaction between yeasts and human DCs does not solely depend on yeast viability, a concept which may suggest a need for further classifications beyond the current definition of a probiotic.

## Introduction

The mucosal-associated lymphoid tissues lining the human gastrointestinal tract contain a network of immune cells with the important task of distinguishing potentially dangerous antigens from harmless substances. Dendritic cells (DCs) govern the balance between immunity and tolerance by sampling of intestinal contents and initiating appropriate immune responses to luminal antigens through pattern recognition receptor signaling, cytokine secretion, and their ability to migrate and present antigen to naïve T cells in draining lymph nodes [1], [2]. At homeostasis, DCs in the intestinal mucosa are conditioned by commensal microorganisms to promote proliferation of Foxp3^+^ regulatory T cells (T_regs_), strong producers of anti-inflammatory IL-10 contributing to intestinal tolerance [1], [3], [4]. During infection or active inflammation, pathogenic microorganisms bind to pattern recognition receptors expressed by DCs and activate signaling pathways involving MAP kinases and the nuclear transcription factor NFκB resulting in production and secretion of a wide range of chemokines and cytokines with distinct inflammatory effects. In this context, DC secretion of inflammatory cytokines such as TNFα and IL-1β is central for acute, innate inflammatory responses involving attraction of neutrophils and macrophages to the site of infection. In addition, DCs are central players in the regulation of adaptive immune responses. For example, DC secretion of IL-12 and IL-6 promotes the proliferation of Th1 and Th17 subpopulations, respectively, whereas DC modulation toward an IL-10 secreting phenotype contributes to induction of T_reg_ responses promoting intestinal tolerance [5], [6]. Furthermore, efficient antigen presentation relies upon DC maturation, a process involving upregulation of co-stimulatory surface molecules as well as modulation of chemokine receptor expression.

Probiotics are live microorganisms which when administered in adequate amounts confer a health benefit on the host [7]. Based on their role as key regulators of intestinal inflammation, DC involvement in probiotic functionality has been studied extensively [8]–[11]. The concept of individual commensal microorganisms influencing the makeup of intestinal T cell subsets via interactions with DCs appears to constitute the foundation for immunoregulatory effects of probiotics [4], and several studies have reported probiotic strains resulting in reduction of intestinal inflammation through modulation of DC function [8], [9], [12]–[14]. Consequently, modulation of DC cytokine secretion and maturation by various microorganisms has elucidated species and strain specific effects that have guided the selection of novel probiotic strains for further investigation [15]–[17].

Although the gut microbiota is dominated by bacteria [18], communities of eukaryotic microorganisms are part of the human microbiome [19]–[21]. In addition, eukaryotes such as food-related yeasts have been utilized for the production of fermented food and beverages and consumed by humans for centuries [22]. Thus, much like for prokaryotes, interactions between eukaryotic microorganisms and the intestinal immune system may influence human health in various ways. While the majority of probiotic microorganisms studied to date are lactic acid producing bacteria, research in yeasts with potentially beneficial influences on human health has mainly revolved around *Saccharomyces boulardii* [23], [24], a yeast taxonomically acknowledged as belonging to the *S. cerevisiae* species [25], [26] but in the following text referred to as *S. boulardii*.

*S. boulardii* has been included in numerous randomized controlled trials and strong clinical evidence exists for the use of *S. boulardii* for the prevention of antibiotic associated diarrhea, Traveler’s diarrhea, and acute infectious diarrheas [27], [28]. In addition, *S. boulardii* has shown a positive impact on disease outcome in clinical studies of inflammatory bowel diseases such as Crohn’s disease and ulcerative colitis [27], indicating an ability of *S. boulardii* to influence human immune responses underlying intestinal inflammation. The molecular basis for the beneficial effects of *S. boulardii* has been subject to extensive study, *in vitro* as well as in animal models, and *S. boulardii* has been found to impact inflammatory cytokine production by intestinal epithelial cells [29]–[34], peripheral blood mononuclear cells (PBMCs) [24], and DCs [23], [35]–[37], reducing inflammatory scores in experimental colitis models in rodents [24], [30], [32], [33], [38]–[40].

Consequent to the intense research focus on *S. boulardii* as the fundamental probiotic yeast, very little is known about hundreds of non-*Saccharomyces* yeasts in terms of their interaction with the human gastrointestinal immune system. Other food-related yeast species typically associated with dairy products such as kefir and traditional cheeses include *Kluyveromyces lactis*, *Kluyveromyces marxianus*, and *Debaryomyces hansenii* [41]. While isolates of all three species have been evaluated for potential probiotic properties in *in vitro* experimental conditions assessing acid and bile survival, and adhesion to and modulation of cytokine secretion from intestinal epithelial cells [41]–[46], studies of the interactions between these yeasts and specialized immune cells have been far fewer [47], [48].

The current definition of probiotics as “live microorganisms which when administered in adequate amounts confer a health benefit on the host” places importance on the viability of probiotic microorganisms at the site of action, presumably the lower small intestines. This has led to numerous studies focusing on the ability of potentially probiotic microorganisms to survive the harsh conditions of the human gastrointestinal tract, i.e. the acidic environment in the gastric sac and the presence of bile salts in the proximal small intestines [41], [42], [47]. However, while probiotic effects caused by actively secreted molecules will depend on a probiotic microorganism being alive [23], [30], [34], [35], other probiotic effects may depend solely on the interaction of microbial cell wall molecules and surface receptors expressed by host cells without the need for an active metabolic function of the probiotic. Indeed, several studies have found heat killed, UV irradiated, and live bacteria to display equal DC stimulatory patterns *in vitro* [6], [10], [49]–[52]. Others have described the failure of nonviable *Saccharomyces* yeasts to prevent pathogen induced cytokine and chemokine expression in cultured epithelial cells [29], [43], while a third study reported that *S. boulardii* maintained an inhibitory effect on *Salmonella* induced signaling pathways in epithelial cells even after being subjected to a membrane disrupting glass bead treatment, thus indicating the likely importance of yeast cell wall structures for the observed inhibition [32].

Multiplexed immunoassays based on the principles of flow cytometry allow for simultaneous determination of numerous soluble proteins in very small sample volumes. The combination of high throughput and impressive accuracy, sensitivity, and reproducibility make these experimental techniques highly relevant for screening purposes where rapid quantification of multiple compounds is critical [53], [54].

The aim of the present study was to evaluate a broad spectrum of yeasts (170 strains representing 75 diverse yeast species were included in the study) for modulation of inflammatory cytokine secretion by human DCs, as compared to cytokine responses induced by a *S. boulardii* (Ultra-Levure) reference strain with probiotic properties documented in clinical trials [27]. To our knowledge, this is the first large-scale study of highly diverse yeasts in terms of their modulation of DC function, incorporating secretion levels of several cytokines. Furthermore, we investigated whether cytokine inducing interactions between yeasts and human DCs are dependent upon yeast viability or rather a product of membrane interactions regardless of yeast metabolic function.

## Materials and Methods

### Yeast strains and growth conditions

Yeast strains included in this study were obtained from CBS (www.cbs.knaw.nl). 170 strains were selected based on a desire to include a broad range of yeast biodiversity (see complete list of included strains in Table 1). Strains were cultured in YPD media (0,5 % yeast extract, 1 % peptone, 1,1 % D-glucose) at 30°C under aerobic conditions. Early stationary growth phase yeast cultures were harvested by centrifugation, washed twice with DC media (RPMI 1640 supplemented with 10 mM HEPES (Sigma-Aldrich, Schnelldorf, Germany) and 50 µM 2-mercaptoethanol (Sigma-Aldrich, Schnelldorf, Germany)), OD adjusted in DC media containing 10 % glycerol, and cryopreserved at −80°C until time of DC stimulation. Viability of frozen yeast cultures was verified by staining with propidium iodide. For some experiments, yeast strains were UV irradiated (70,000 µJ/cm^2^ for 5 min) or heat treated (80°C at 650 rpm for 5 min) prior to cryopreservation at −80°C. Yeast cell viability after UV irradiation or heat treatment was assessed by propidium iodide staining (UV <40% intact cells; heat <20% intact cells), and the reproductive ability of UV irradiated and heat treated yeasts was determined by colony counts after 48 h incubation of YPD agar plates at 30°C.

**Table 1:** Yeast strains included in study.

Genus	Species	Strains
*Ambrosiozyma*	*monospora*	CBS2554
*Barnettozyma*	*pratensis*	CBS9053, CBS9055
*Bensingtonia*	*yamatoana*	CBS9336
*Botryozyma*	*mucatilis*	CBS9042, CBS9043
*Brettanomyces*	*custersianus*	CBS4806, CBS5207, CBS5208
	*naardenensis*	CBS7540
*Candida*	*amphixiae*	CBS9877
	*anneliseae*	CBS9837
	*atakaporum*	CBS9833
	*athensensis*	CBS9840, CBS9841
	*blattae*	CBS9871
	*bohiensis*	CBS9897
	*bolitotheri*	CBS9832
	*bombi*	CBS9017
	*buenavistaensis*	CBS9895
	*choctaworum*	CBS9831
	*chrysomelidarum*	CBS9904
	*elateridarum*	CBS9842
	*ghanaensis*	CBS8798
	*gigantensis*	CBS9896
	*litsaeae*	CBS8799
	*michaelii*	CBS9878
	*palmioleophila*	CBS8109
	*powellii*	CBS8795
	*taliae*	CBS9838
*Citeromyces*	*siamensis*	CBS9152, CBS9153
*Cryptococcus*	*laurentii* var. *laurentii*	CBS8796
	*podzolicus*	CBS9357, CBS9358
*Cryptotrichosporon*	*anacardii*	CBS9549, CBS9551
*Debaryomyces*	*fabryi*	CBS10579
	*hansenii*	CBS116, CBS767, CBS773, CBS1101, CBS1119, CBS1121, CBS1123, CBS1129, CBS1519, CBS1791, CBS1795, CBS1962, CBS2331, CBS2333, CBS4890, CBS5139, CBS5140, CBS6089, CBS6574, CBS7032, CBS7848, CBS8339, CBS9682, CBS9685, CBS9696
	*subglobosus*	CBS792, CBS1128
*Dekkera*	*anomala*	CBS77, CBS4212, CBS4711, CBS7250, CBS8138
	*bruxellensis*	CBS72, CBS75, CBS96, CBS2547, CBS4459, CBS4482, CBS4601, CBS4602, CBS6055
*Geotrichum*	*cucujoidarum*	CBS9893
*Hanseniaspora*	*lachancei*	CBS8818, CBS8819
	*opuntiae*	CBS8820, CBS9791
*Kazachstania*	*exigua*	CBS9330
*Kluyveromyces*	*lactis* var. *drosophilarum*	CBS9056
	*lactis* var. *lactis*	CBS9057, CBS9058, CBS9059, CBS9060
	*marxianus*	CBS1553
*Kurtzmaniella*	*cleridarum*	CBS8793
*Lachancea*	*fermentati*	CBS797
	*kluyveri*	CBS6545, CBS6546, CBS6547
	*thermotolerans*	CHCC5756
*Lodderomyces*	*elongisporus*	CBS7803
*Metschnikowia*	*arizonensis*	CBS9064
	*borealis*	CBS8431, CBS8432
	*gruessii*	CBS9029, CBS9030
	*koreensis*	CBS9066
	*kunwiensis*	CBS9067, CBS9677, CBS9679, CBS9681
	*noctiluminum*	CBS9907
	*reukaufii*	CBS9018, CBS9019, CBS9020, CBS9021, CBS9022
*Naumovozyma*	*castelli*	CBS2248, CBS4310, CBS4906
	*dairensis*	CBS421
*Ogataea*	*dorogensis*	CBS9260, CBS9261
*Pichia*	*kluyveri*	CHCC11259
	*mandshurica*	CBS209
	*myanmarensis*	CBS9786
	*sporocuriosa*	CBS9200
*Rhodosporidium*	*diobovatum*	CBS9081, CBS9084
	*sphaerocarpum*	CBS9080
	*toruloides*	CBS14
*Rhodotorula*	*mucilaginosa* var. *mucilaginosa*	CBS9070, CBS9078, CBS9083
*Saccharomyces*	*arboricolus*	CBS10644
	*bayanus*	CBS381, CBS1641, CBS9787
	*boulardii*	CHCC11905, CHCC11906, 259*, 7103*, 7135*, 7136*, LSB*, Sb.A*, Sb.L*, Sb.P*
	*cariocanus*	CBS5313, CBS7994, CBS8841
	*cerevisiae*	CBS1646, CHCCJ4848, CBS6128, CHCC7036, CBS9564
	*kudriavzevii*	CBS8840
	*mikatae*	CBS8839, CBS10522, CBS10523
	*paradoxus*	CBS8442
	*pastorianus*	CBS1462, CBS1642
*Zygosaccharomyces*	*mellis*	CBS711, CBS738
	*rouxii*	CBS708, CBS733

Strain sources: CBS Centraalbureau voor Schimmelcultures, Utrecht, The Netherlands

CHCC Chr. Hansen Culture Collection, Hørsholm, Denmark

* van der Aa Kühle, A., Jespersen, L., 2003. Systematic and Applied Microbiology 26, 564–571.

### Monocyte-derived DC generation

Immature monocyte-derived DCs were generated *in vitro* by a 6 day procedure as described [50]. Human buffy coats from healthy donors were supplied by Department of Clinical Immunology at Copenhagen University Hospital, Copenhagen, Denmark. Use of human samples with no identifying information was approved by The National Committee on Health Research and the Danish Society for Clinical Immunology, and all donors gave informed written consent upon donation. Briefly, human peripheral blood mononuclear cells were obtained from buffy coats by density gradient centrifugation using Ficoll-Paque PLUS (GE Healthcare, Freiburg, Germany). Monocytes were isolated by positive selection for CD14 using magnetic-activated cell sorting with CD14 microbeads (Miltenyi Biotec, Bergisch Gladbach, Germany) and cultured at a density of 2×10^6^ cells/mL in complete DC media (RPMI 1640 supplemented with 10 mM HEPES (Sigma-Aldrich, Schnelldorf, Germany), 50 µM 2-mercaptoethanol (Sigma-Aldrich, Schnelldorf, Germany), 2 mM L-glutamine (Life Technologies Ltd, Paisley, UK), 10 % heat-inactivated fetal bovine serum (Invitrogen, Paisley, UK), 100 U/mL penicillin (Biological Industries, Kibbutz Beit-Haemek, Israel), and 100 µg/mL streptomycin (Biological Industries, Kibbutz Beit-Haemek, Israel)) containing 30 ng/mL human recombinant IL-4 and 20 ng/mL human recombinant GM-CSF (both from Sigma-Aldrich, Saint Louis, USA) at 37°C, 5 % CO_2_. Fresh complete DC media containing full doses of IL-4 and GM-CSF was added after three days of culture. At day 6, differentiation to immature DCs was verified by surface marker expression analysis (CD11c >90% expression; CD1a >75% expression).

### DC stimulation

Immature DCs were resuspended in fresh complete DC media containing no antibiotics, seeded in 96-well plates at 1×10^5^ cells/well, and allowed to acclimate at 37°C, 5 % CO_2_, for at least one hour before stimulation. DC stimulation using thawed yeast strains was performed at a yeast:DC ratio of 10∶1, and stimulated DCs were incubated for 20 h at 37°C, 5 % CO_2_, as time-course experiments had shown a 20 h stimulation time to result in quantifiable levels of all cytokines of interest. After 20 h stimulation, DC supernatants were sterile filtered through a 0.2 µm AcroPrep Advance 96-well filter plate (Pall Corporation, Ann Arbor, MI, USA) and stored at −80°C until time of cytokine quantification.

### DC staining for quantification of co-stimulatory molecules and chemokine receptors

Immediately following 20 h stimulation time, DCs were collected, centrifuged at 200x g for 5 min, and resuspended in cold PBS containing 2 % BSA. Staining was performed using the following monoclonal antibodies: FITC-conjugated anti-human CD80 (clone L307.4), FITC-conjugated anti-human CD86 (clone 2331), APC-conjugated anti-human CCR6 (clone 11A9), FITC-conjugated anti-human CCR7 (clone 150503), and appropriate isotype controls (all from BD Biosciences, Erembodegem, Belgium). DCs were incubated with mAb for 30 min on ice protected from light, followed by repeated wash steps using 1 mL cold PBS 2 % BSA. Finally, DCs were resuspended in PBS 2 % BSA and kept on ice until flow cytometric analysis. Samples were acquired on an LSRFortessa flow cytometer (BD Biosciences, San Jose, CA, USA) using FACSDiva software (BD Biosciences, San Jose, CA, USA).

### Cytokine quantification

Secreted levels of IL-12, TNF, IL-10, IL-6, and IL-1β were quantified by the Human Inflammatory Cytokines cytometric bead array (CBA) kit (BD Biosciences, Erembodegem, Belgium) according to the manufacturer’s instructions. Briefly, fluorescent beads coated with monoclonal capture antibodies were mixed with PE conjugated detection antibodies and recombinant standards or test samples and allowed to form sandwich complexes during 3 h incubation protected from light. After repeated wash steps, samples were acquired on an LSRFortessa flow cytometer (BD Biosciences, San Jose, CA, USA) and data analysis was performed using the FCAP Array 3 software (BD Biosciences, San Jose, CA, USA). Detection limits for individual cytokines were as follows: 1.9 pg/mL IL-12, 3.7 pg/mL TNF, 3.3 pg/mL IL-10, 2.5 pg/mL IL-6, and 7.2 pg/mL IL-1β.

### Multivariate data analysis and statistical analysis

Multivariate data analysis was performed using SIMCA-P+ 13 (Umetrics, Umeå, Sweden). A PCA-class model was generated based on data for induced levels of IL-12, IL-10, IL-6, TNFα, and IL-1β by 12 biological replicates of the *S. boulardii* reference strain, thereby centering the plot point of origin on the cytokine profile induced by the *S. boulardii* reference strain. Induced cytokine data (IL-12, IL-10, IL-6, TNFα, and IL-1β) for the 170 yeast strains included in the screen constituted the prediction data set; i.e. the distance of a given yeast strain from the plot point of origin indicates how closely the induced cytokine profile resembles that of the *S. boulardii* reference strain.

Statistical analysis (one-way ANOVA with Bonferroni’s multiple comparison post test) was performed using GraphPad Prism 5 (GraphPad Software, La Jolla, USA).

## Results

### Yeasts induce highly diverse cytokine profiles in human DCs

**Figure 1:**
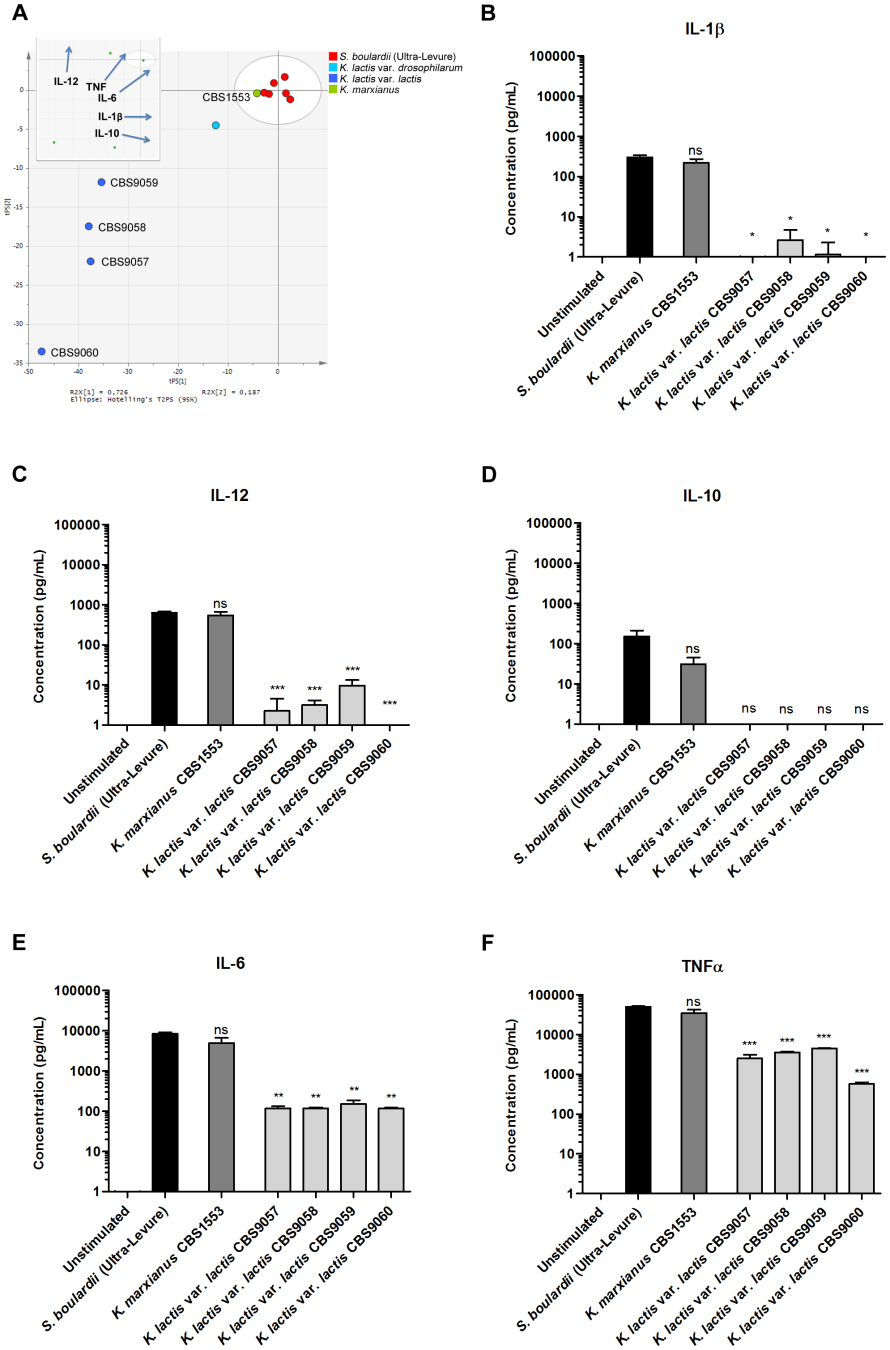
DC cytokine secretion induced by *Kluyveromyces* yeasts. **A.** Principal component analysis (PCA) scatter plot of DC cytokine profiles induced by *Kluyveromyces* yeast strains and the *S. boulardii* reference strain. Each dot represents induced cytokine data (IL-12, IL-10, IL-6, TNFα, and IL-1β) for one yeast strain, entered as the mean value of two biological replicates, and colored according to yeast species. The six red dots representing biological replicates of the *S. boulardii* reference strain show the deviation in the model. The insert represents loadings of individual cytokines included in the PCA model; for example, high IL-12 inducing yeasts are placed high along the plot Y axis, whereas strong IL-10 inducing yeasts are located to the right along the plot X axis. Levels of **B.** IL-1β, **C.** IL-12, **D.** IL-10, **E.** IL-6, and **F.** TNFα secreted by human monocyte-derived DCs following 20 h stimulation with DC media containing 10 % glycerol (unstimulated) or *S. boulardii* (Ultra-Levure), *K. marxianus* (CBS1553), or *K. lactis* var. *lactis* (CBS9057, CBS9058, CBS9059, CBS9060) at a yeast:DC ratio of 10∶1. Data are representative of two independent experiments, error bars represent SEM. One-way ANOVA, Bonferroni’s multiple comparison post test, indicating significant differences from cytokine levels induced by *S. boulardii*. ns, not significant; *, P<0.05; **, P<0.01; ***, P<0.001.

Given that modulation of DC cytokine secretion has been linked to probiotic functionality related to intestinal inflammation, we evaluated yeast modulation of DC secretion of five inflammation related cytokines *in vitro*. We exposed human DCs to each yeast strain in duplicate at a 10∶1 yeast:DC ratio and quantified secreted levels of IL-12, IL-10, IL-6, TNFα, and IL-1β after 20 h stimulation. Stimulation time was selected based on time-course experiments indicating 20 h as ideal for obtaining quantifiable levels of cytokines produced at a slow rate (IL-12, IL-10, and IL-1β) without reaching saturation conditions for rapidly secreted cytokines (IL-6 and TNFα) (Fig S1). As a point of reference, we included a *S. boulardii* strain with probiotic effects documented in clinical trials [27]. As expected, *S. boulardii* engaged human immune cells, as evidenced by induction of a robust response across all five cytokines (Fig 1). In addition, *S. boulardii* induced high levels of the co-stimulatory molecules CD80 and CD86, indicative of strong activation of the immature DCs (Fig S2), and affected DC chemokine receptor expression, as observed by down-regulation of CCR6 and strong up-regulation of CCR7 (Fig S2), indicating that *S. boulardii* activates immature DCs to a mature phenotype primed for lymph node migration and efficient antigen presentation.

**Figure 2:**
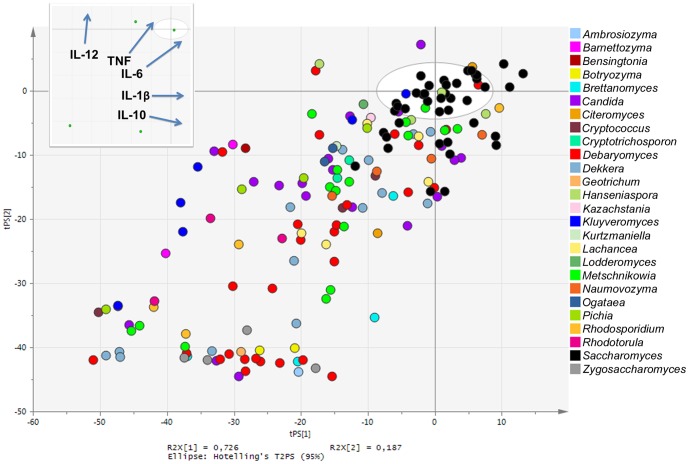
Yeast induced cytokine profiles in human DCs. Principal component analysis (PCA) scatter plot of induced cytokine data for 170 yeast strains included in screen. Each dot represents induced cytokine data (IL-12, IL-10, IL-6, TNFα, and IL-1β) for one yeast strain, entered as the mean value of two biological replicates, and colored according to yeast genera. The plot point of origin is centered on the cytokine profile induced by the *S. boulardii* reference strain, and thus the distance of a given yeast strain from the plot point of origin indicates how closely the induced cytokine profile resembles that of the *S. boulardii* reference strain. The insert represents loadings of individual cytokines included in the PCA model; for example, high IL-12 inducing yeasts are placed high along the plot Y axis, whereas strong IL-10 inducing yeasts are located to the right along the plot X axis.

For comparison of the yeast induced DC cytokine profiles, multivariate data analysis was applied as a valuable tool for visualizing and grouping yeast strains based on quantified levels of all five cytokines. A PCA-class model was generated based on data for induced levels of IL-12, IL-10, IL-6, TNFα, and IL-1β by 12 biological replicates of the *S. boulardii* reference strain, thereby centering the PCA plot point of origin on the cytokine profile induced by the reference strain (Fig 2). Induced cytokine data (IL-12, IL-10, IL-6, TNFα, and IL-1β) for the 170 yeast strains included in the study constituted the prediction data set; i.e. the distance of a yeast strain from the plot point of origin indicates how closely the induced cytokine profile resembles that of the *S. boulardii* reference strain. Visualizing the obtained cytokine profiles in this way revealed the interesting fact that induction of all five cytokines was positively correlated in our study, as shown by the loadings of individual cytokines in the Figure 2 insert.

As displayed in Figure 2, the yeasts included in this study induced highly diverse cytokine profiles in human DCs. Not surprisingly, the majority of *Saccharomyces* yeasts induced cytokine profiles very similar to the *S. boulardii* reference strain, as indicated by their location very close to the plot point of origin in Figure 2. In addition, this overview plot shows *Saccharomyces* yeasts as strong cytokine inducers, with very few non-*Saccharomyces* yeasts inducing cytokine levels higher than the *S. boulardii* reference strain (i.e. not many yeast strains present in the upper right quadrant of the plot). For non-*Saccharomyces* yeasts, we observe a broad range of cytokine inducing properties. For instance, a third of the included *Debaryomyces* strains, several *Dekkera* strains, and all included *Zygosaccharomyces* isolates dominate a distinct cluster of very low cytokine inducing yeasts present at the bottom left corner of the plot (Fig 2).

### Distinct differences observed in cytokine inducing properties of individual yeast genera

Next, we focused on the induced DC cytokine profiles of individual yeast genera. Six *Kluyveromyces* strains representing the species *K. marxianus*, *K. lactis* var. *lactis*, and *K. lactis* var. *drosophilarum* were included in our study, and multivariate data analysis of the induced DC cytokine profiles revealed clear species distinctions in immune stimulating capacities (Fig 1A). *K. marxianus* (CBS1553) induced DC cytokine levels statistically indistinguishable (P>0.05) from the profile induced by the *S. boulardii* reference strain for every one of the quantified cytokines (Fig 1B-F). In contrast, the four *K. lactis* var. *lactis* strains (CBS9057, CBS9058, CBS9059, CBS9060) induced much lower levels of cytokines; in particular, induced levels of IL-12, IL-10, and IL-1β were near or below the detection limit of the assay. Strikingly, no significant differences were observed between the DC cytokine profiles induced by the four *K. lactis* var. *lactis* strains (Fig 1B-F, P>0.05 for all quantified cytokines).

**Figure 3:**
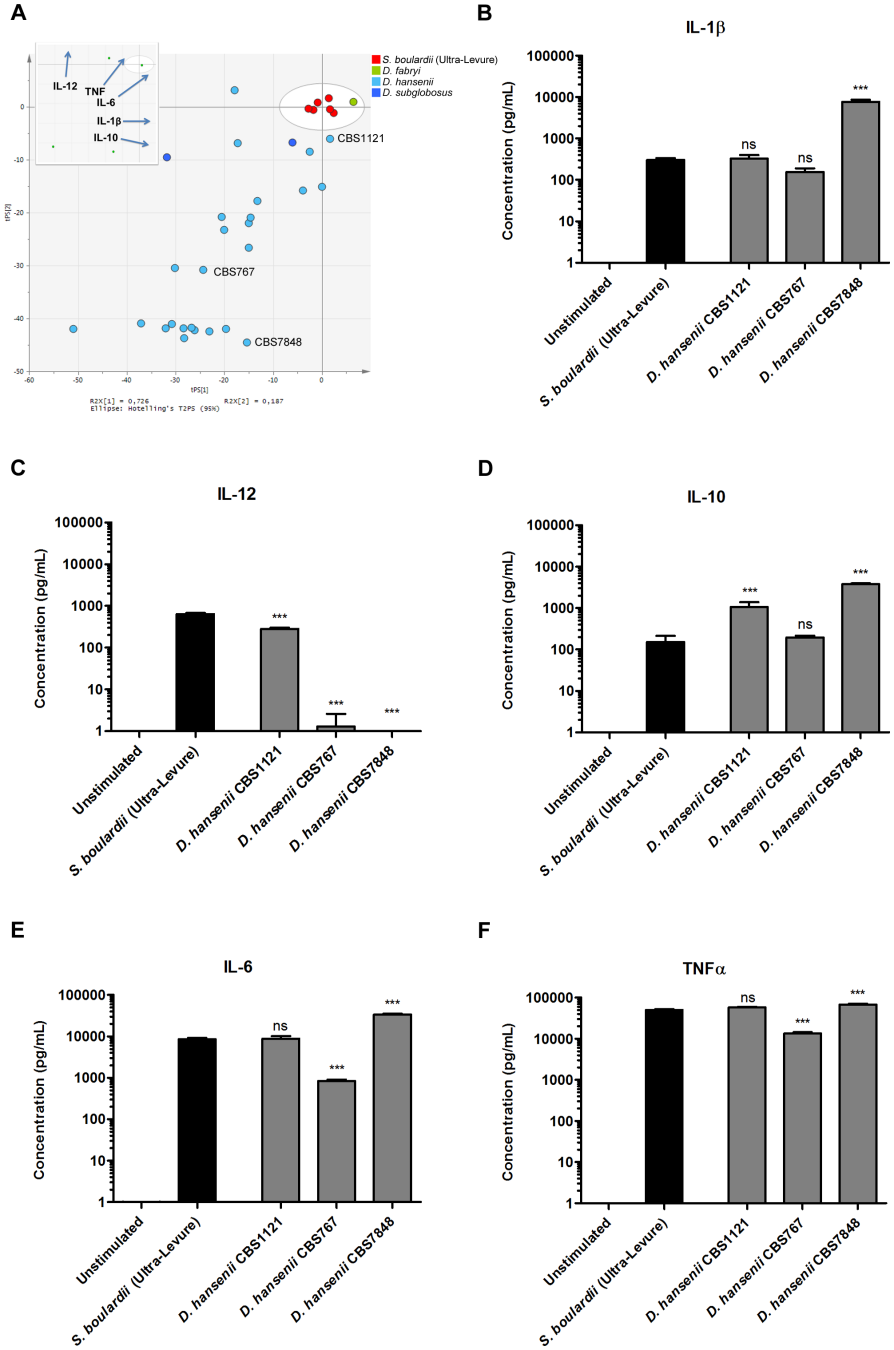
DC cytokine secretion induced by *Debaryomyces* yeasts. **A.** Principal component analysis (PCA) scatter plot of DC cytokine profiles induced by *Debaryomyces* yeast strains and the *S. boulardii* reference strain. Each dot represents induced cytokine data (IL-12, IL-10, IL-6, TNFα, and IL-1β) for one yeast strain, entered as the mean value of two biological replicates, and colored according to yeast species. The six red dots representing biological replicates of the *S. boulardii* reference strain show the deviation in the model. The insert represents loadings of individual cytokines included in the PCA model; for example, high IL-12 inducing yeasts are placed high along the plot Y axis, whereas strong IL-10 inducing yeasts are located to the right along the plot X axis. Levels of **B.** IL-1β, **C.** IL-12, **D.** IL-10, **E.** IL-6, and **F.** TNFα secreted by human monocyte-derived DCs following 20 h stimulation with DC media containing 10 % glycerol (unstimulated), *S. boulardii* (Ultra-Levure), or *D. hansenii* (CBS1121, CBS767, CBS7848) at a yeast:DC ratio of 10∶1. Data are representative of two independent experiments, error bars represent SEM. One-way ANOVA, Bonferroni’s multiple comparison post test, indicating significant differences from cytokine levels induced by *S. boulardii*. ns, not significant; *, P<0.05; **, P<0.01; ***, P<0.001.

For *Debaryomyces* yeasts, 25 of the 28 strains included in our study represented the species *D. hansenii*, and the induced DC cytokine profiles revealed a remarkable diversity in immune stimulating properties (Fig 3A). The strain CBS1121 induced DC cytokine levels similar to the *S. boulardii* reference strain (Fig 3B-F), as indicated by secreted levels of IL-1β, IL-6, and TNFα being statistically indistinguishable from *S. boulardii* induced levels. The *D. hansenii* type strain (CBS767) displayed much poorer cytokine induction capabilities, as seen by significantly lower induction of the pro-inflammatory cytokines IL-12, IL-6, and TNFα. Finally, the *D. hansenii* strain CBS7848 induced a DC cytokine profile characterized by levels of IL-1β, IL-10, IL-6, and TNFα significantly higher than the *S. boulardii* reference strain, yet failed to induce detectable levels of IL-12.

**Figure 4:**
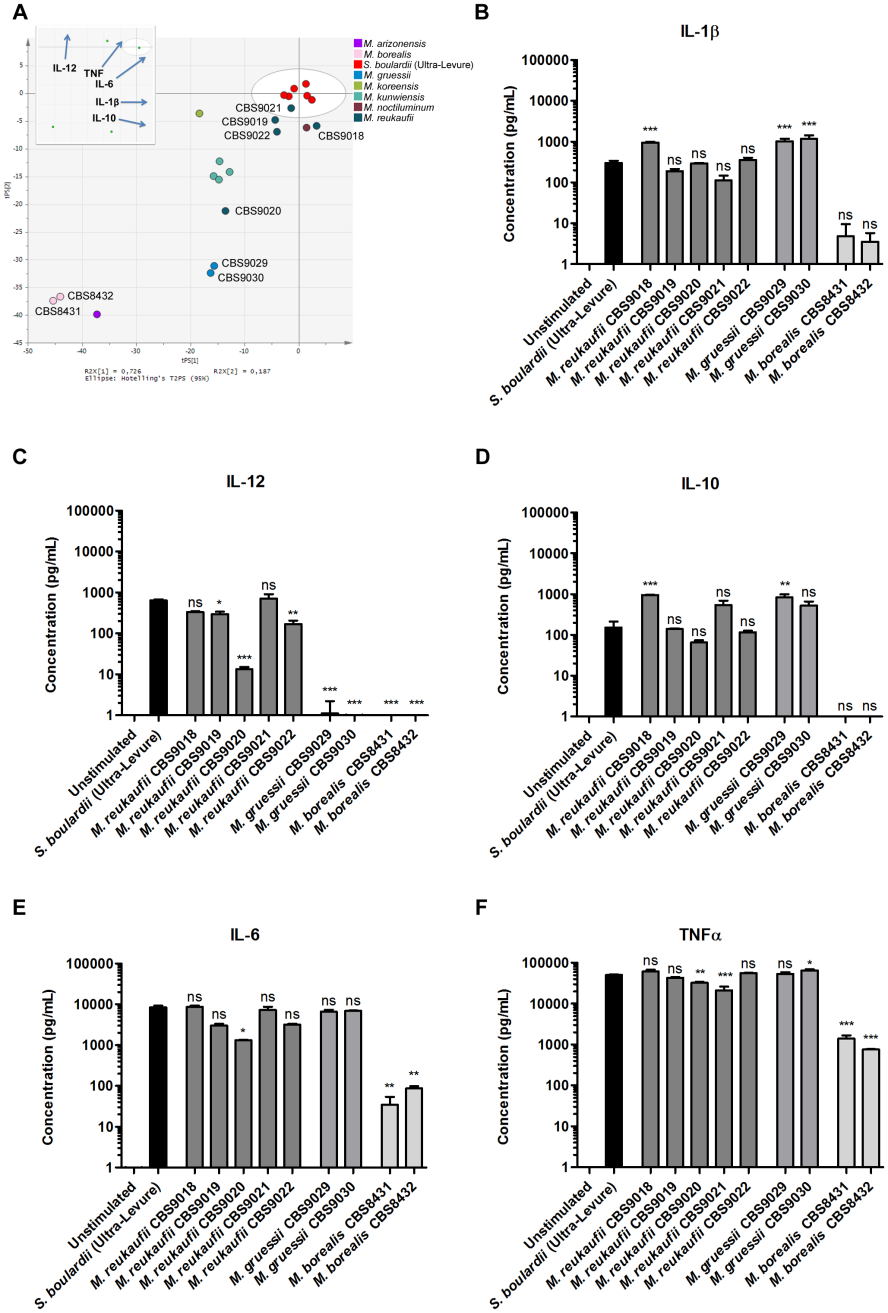
DC cytokine secretion induced by *Metschnikowia* yeasts. **A.** Principal component analysis (PCA) scatter plot of DC cytokine profiles induced by *Metschnikowia* yeast strains and the *S. boulardii* reference strain. Each dot represents induced cytokine data (IL-12, IL-10, IL-6, TNFα, and IL-1β) for one yeast strain, entered as the mean value of two biological replicates, and colored according to yeast species. The six red dots representing biological replicates of the *S. boulardii* reference strain show the deviation in the model. The insert represents loadings of individual cytokines included in the PCA model; for example, high IL-12 inducing yeasts are placed high along the plot Y axis, whereas strong IL-10 inducing yeasts are located to the right along the plot X axis. Levels of **B.** IL-1β, **C.** IL-12, **D.** IL-10, **E.** IL-6, and **F.** TNFα secreted by human monocyte-derived DCs following 20 h stimulation with DC media containing 10 % glycerol (unstimulated) or *S. boulardii* (Ultra-Levure), *M. reukaufii* (CBS9018, CBS9019, CBS9020, CBS9021, CBS9022), *M. gruessii* (CBS9029, CBS9030), or *M. borealis* (CBS8431, CBS8432) at a yeast:DC ratio of 10∶1. Data are representative of two independent experiments, error bars represent SEM. One-way ANOVA, Bonferroni’s multiple comparison post test, indicating significant differences from cytokine levels induced by *S. boulardii*. ns, not significant; *, P<0.05; **, P<0.01; ***, P<0.001.

The yeast genus *Metschnikowia* represents a large family of yeasts which, to the best of our knowledge, has not been explored for properties relating to human health. The PCA plot in Figure 4A displays the DC cytokine profiles induced by the 16 isolates representing seven *Metschnikowia* species included in our study. The plot reveals striking species distinctions separating species with highly diverse cytokine inducing properties. While isolates of *M. reukaufii* induced DC cytokine profiles very similar to the *S. boulardii* reference strain across all five cytokines, *M. gruessii* isolates induced robust levels of IL-1β, IL-10, IL-6, and TNFα, yet undetectable levels of IL-12 (Fig 4B-F). In contrast, *M. borealis* displayed poor cytokine inducing properties in general, as seen by an induced DC cytokine profile characterized by significantly lower cytokine levels compared to the *S. boulardii* reference strain.

### Yeasts are capable of DC stimulation independently of viability

**Figure 5:**
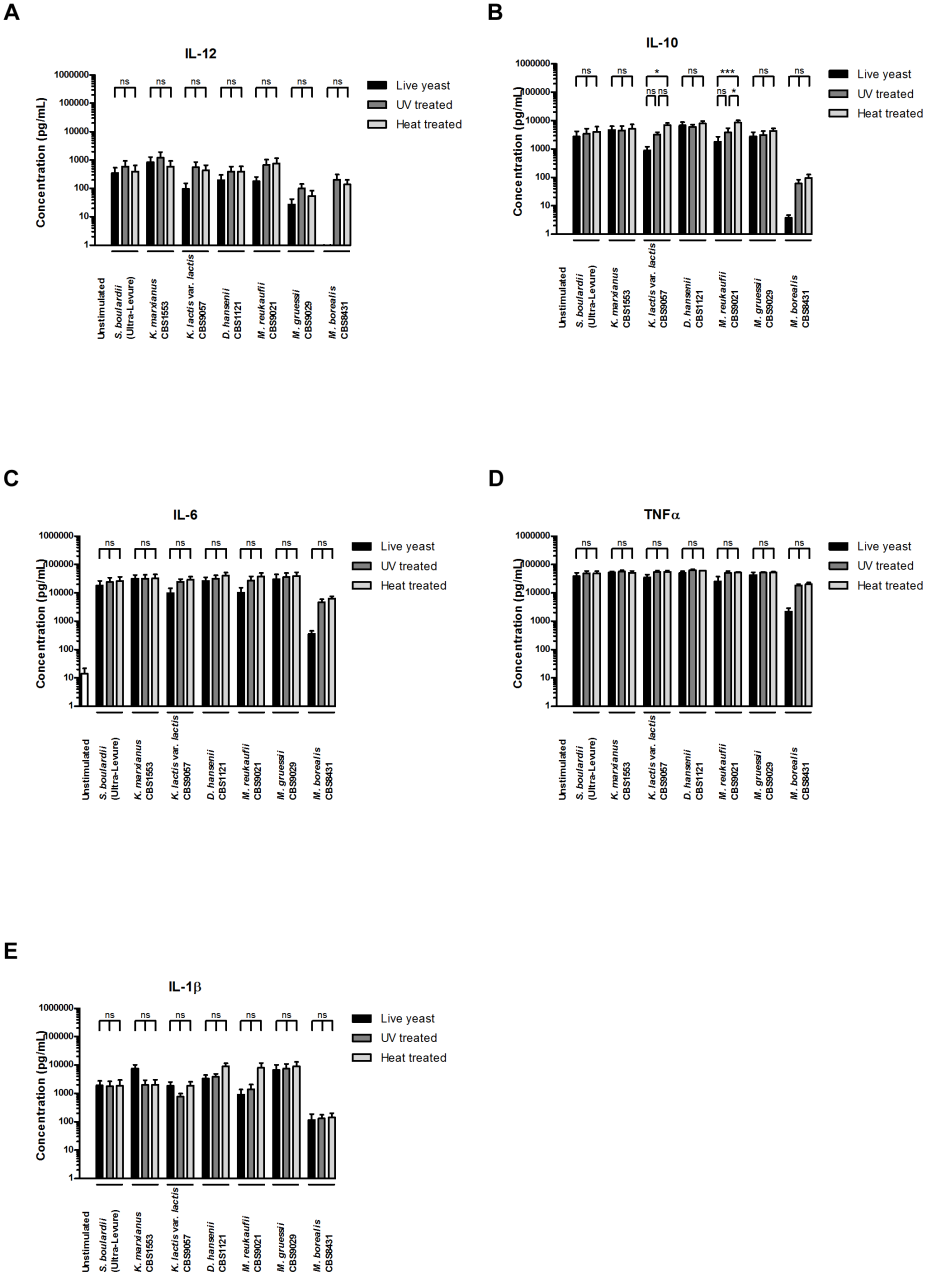
Yeast induced DC cytokine secretion occurs independently of yeast metabolic function. Levels of **A.** IL-12, **B.** IL-10, **C.** IL-6, **D.** TNFα, and **E.** IL-1β secreted by human monocyte-derived DCs following 20 h stimulation with DC media containing 10 % glycerol (unstimulated) or *S. boulardii* (Ultra-Levure), *K. marxianus* (CBS1553), *K. lactis* var. *lactis* (CBS9057), *D. hansenii* (CBS1121), *M. reukaufii* (CBS9021), *M. gruessii* (CBS9029), or *M. borealis* (CBS8431). For each yeast strain, DC stimulation was performed with live yeast, UV irradiated yeast, and heat treated yeast at a yeast:DC ratio of 10∶1. Data are expressed as mean±SEM (n = 4). One-way ANOVA, Bonferroni’s multiple comparison post test, indicating significant differences between cytokine levels induced by live, UV irradiated, and heat treated yeast for each strain. ns, not significant; *, P<0.05; **, P<0.01; ***, P<0.001.

Next, we investigated whether the observed interactions between yeasts and DCs were dependent upon yeast viability. We hypothesized that yeasts would be able to induce DC activation regardless of metabolic activity and conducted experiments to compare DC stimulation with live, UV irradiated, and heat treated yeast. UV irradiation conditions were designed to generate relatively intact yeast cells unable to reproduce, whereas heat treatment was intended to cause severe yeast cell membrane disruption. Propidium iodide staining confirmed a reduction in the proportion of intact yeast cells to levels below 40% after UV irradiation and below 20% after heat treatment, and the reproductive inability of UV irradiated and heat treated yeasts was verified by colony counts (data not shown). As presented in Figure 5, the obtained data show no significant differences in cytokine inducing properties between live, UV irradiated, and heat treated *S. boulardii*. Additionally, UV irradiated and heat treated *S. boulardii* were as effective as live yeast in inducing DC co-stimulatory functions and altering chemokine receptor expression levels (Fig S2). Regardless of UV irradiation or heat treatment, *S. boulardii* induced high levels of the co-stimulatory molecules CD80 and CD86, and modulated DC chemokine receptor expression, as observed by down-regulation of CCR6 and strong up-regulation of CCR7 (Fig S2).

For non-*Saccharomyces* yeasts, we observed a remarkably similar pattern. *K. marxianus* induced DC secretion of IL-12, IL-10, IL-6, TNFα, and IL-1β was unaffected by UV irradiation or heat treatment of the yeast (Fig 5). In contrast, the ability of *K. lactis* var. *lactis* (CBS9057) to induce IL-10 appeared slightly stronger after heat treatment, whereas induced levels of IL-12, IL-6, TNFα, and IL-1β remained unchanged. *D. hansenii* (CBS1121) induced cytokine secretion was unaffected by UV irradiation as well as heat treatment (Fig 5). For *Metschnikowia* yeasts, we observed differential modification of cytokine inducing properties between the three species examined. Whereas *M. gruessii* and *M. borealis* were unaffected by UV irradiation as well as heat treatment, the IL-10 inducing properties of *M. reukaufii* appeared slightly potentiated by the heat treatment (Fig 5).

## Discussion

The complex interplay between the gut microbiota and the intestinal immune system as this relates to human health and disease has received increasing attention recently [3], [4], [19]. Novel understandings of how the composition of resident populations of intestinal microorganisms can significantly impact human health in areas as diverse as obesity, allergy, and inflammatory disorders, is making this an area of intense research [19]. Maintenance of intestinal homeostasis requires a carefully regulated network of immune cells responsible for controlling the delicate balance between populations of potentially pro-inflammatory effector cells such as Th1 and Th17 cells and anti-inflammatory T_reg_ cells [4], and intestinal DCs play a central role in orchestrating both innate and adaptive immune responses to commensal microorganisms as well as invasive pathogens [1], [10].

As research in yeasts with a potentially beneficial impact on human health has focused almost exclusively on *S. boulardii*, the aim of the present study was to evaluate immune modulation by a large number of highly diverse yeasts in order to build a data set to serve as a foundation for selecting strains for further mechanistic studies. Choosing an experimental system involving human monocyte-derived DCs resulted in the necessary throughput and by employing a multiplexed immunoassay and multivariate data analysis to incorporate data for yeast induced DC secretion of five cytokines with distinct inflammatory effects we were able to assess the modulation of DC cytokine secretion by 170 yeast strains representing a broad spectrum of biodiversity. In contrast to studies focusing on modulation of a challenge induced DC cytokine response [23], [36], our experimental setup evaluated the ability of yeasts to engage DCs *per se*.

The ability of *S. boulardii* to engage human immune cells and influence cytokine secretion *in vitro* is well established and has been linked to improved inflammatory scores in rodent colitis models. Likewise, commensal bacterial strains have been found to induce strong DC maturation and inflammatory cytokine secretion, yet display tolerogenic properties promoting T cell hyporesponsiveness [10], and strains with known probiotic properties have been shown to induce transient pro-inflammatory host responses in rodents upon initial colonization [55]. Accordingly, we observed robust induction of DC cytokines when human DCs were cultured in the presence of a *S. boulardii* reference strain with probiotic properties documented in clinical trials [27], indicating the expected ability of *S. boulardii* to engage human DCs and impact their secretion of cytokines with distinct inflammatory effects. In addition, we found the *S. boulardii* reference strain capable of inducing co-stimulatory functions and modulating chemokine receptor expression towards an activated DC phenotype primed for lymph node migration and efficient antigen presentation. These findings are supported by reports of *S. boulardii* modulating LPS induced CD80 and CCR7 expression [23], [35], and echo reports of commensal bacterial strains inducing DC maturation, as indicated by increased surface expression of CD80, CD86, and CCR7 [8], [10]. Our observation that yeasts belonging to the *Saccharomyces* genus are among the strongest cytokine inducing yeasts included in the present study, and that all included *Saccharomyces* yeasts induce DC cytokine profiles very similar to that induced by the *S. boulardii* reference strain, parallels the finding of a recent study where six live yeast strains representing the species *S. bayanus*, *S. cerevisiae*, and *S. pastorianus* induced non-discriminatory cytokine profiles in human PBMCs [24].

The remarkable diversity in cytokine inducing properties observed for non-*Saccharomyces* yeasts reflects the high diversity among the included yeast isolates. As the selection criteria for inclusion in the present study were based on a desire to include a broad representation of yeast biodiversity and thus had no apparent link to properties of immune modulation, it is not surprising that while some non-*Saccharomyces* yeasts exhibit strong cytokine inducing properties, others present as far more immunologically inert.

It is generally accepted that probiotic properties of bacteria are not only species but also strain dependent [56]–[58]. Interestingly, whereas our data agree with this notion for a number of included yeast species, other yeast genera display distinct species clustering in DC cytokine inducing properties.

The genus *Kluyveromyces* includes food-related yeasts typically isolated from fermented dairy products, and the health-promoting effects associated with the consumption of these products have led to several studies investigating individual isolates for properties related to human health. A recent study evaluating the immune modulatory properties of a *K. marxianus* strain *in vitro* found a significant impact on the cytokine secretion of IL-6, TNFα, and IL-1β by human PBMCs [47]. While the *K. marxianus* isolate included in the present study is not identical, this does support our observation that *K. marxianus* induces a cytokine profile statistically indistinguishable from that of the *S. boulardii* reference strain, clearly indicating the ability of *K. marxianus* to engage specialized immune cells and impact the secretion of cytokines with distinct effects on adaptive immune responses. We observed distinct species differences clearly separating *K. marxianus* and *K. lactis* var. *lactis* in terms of their ability to induce DC cytokine secretion, with all four *K. lactis* var. *lactis* strains displaying an apparent inability to induce significant levels of cytokines. This is supported by a study finding *K. lactis* unable to induce detectable levels of the cytokines IL-6 and TNFα in a system evaluating interactions between yeast and cultured intestinal epithelial cells [44]. As the same study found *K. lactis* yeasts able to stimulate IL-8 secretion, our data may reflect differences between yeasts mainly interacting with highly abundant epithelial cells triggering innate immune mechanisms such as neutrophil and monocyte recruitment and yeasts capable of engaging specialized immune cells playing an active role in adaptive immune responses.

As discussed above, the species distinction in DC cytokine inducing properties is unmistakable for *Kluyveromyces* yeasts included in the present study. In contrast, the included isolates representing another genus comprising many food-related yeasts, namely *Debaryomyces*, display highly diverse and strain dependent DC inducing properties. Despite being highly abundant in nature and well known as food-related yeasts typically isolated from dairy, meat, and fermented soy products [59], all of which suggest likely human contact with these microorganisms, *Debaryomyces* yeasts have not been thoroughly studied for properties relating to human health.

The aim of the present study was to provide a first step in a search for novel yeasts with the ability to impact human health through interactions with the intestinal immune system, and naturally, safety constitutes an important factor when considering novel microorganisms with the potential for development of products aimed at human consumption. Consequently, focusing further studies solely on yeast species approved by the European Food Safety Authority for holding qualified presumption of safety (QPS) becomes an attractive proposition. This would suggest an emphasis on *K. marxianus*, *K. lactis*, and *D. hansenii*, all of which hold QPS status [60] and, in the present study, displayed DC cytokine induction properties worthy of further investigation. However, limiting future efforts to already well-known food-related yeasts would present the inherent risk of missing less-studied yeasts with potentially superior properties. The yeast genus *Metschnikowia*, comprising a large family of yeasts primarily isolated from flowers and bees across the European continent, provides an example. To the best of our knowledge, *Metschnikowia* yeasts have not been subject to studies relating to human health, yet the numerous isolates included in our study exhibited a remarkable species distinction in DC cytokine inducing properties warranting further examination.

While the experimental conditions in this study were intended to simulate the *in vivo* situation where microorganisms encounter mucosal DCs during passage through the human gut; naturally, some limitations need to be kept in mind when interpreting the data. Notably, single strain stimulation of DCs may be a necessity for data analysis when evaluating numerous strains but it fails to account for interactions between different members of the intestinal flora. Given the complexity of the human microbiota, the likely interactions between yeasts and other microorganisms present in the intestinal tract may result in different DC cytokine profiles. This highlights the importance of extending the current knowledge of select strains through additional studies incorporating a more complex mix of microorganisms resembling that of the human gastrointestinal tract.

From an industrial point of view, it is of importance to know whether dead yeast cells can be used in the same manner as viable cells. Additionally, yeast cells present in the human gastrointestinal tract may be either viable or dead, which makes it relevant to investigate whether the immunological responses are the same. Further, as yeast cell wall composition and structure are known to vary depending on yeast growth phase and any treatment, the prospect that cell wall structures of nonviable yeast cells may differ from those of viable yeast cells and in turn potentially trigger a different immunological response, led us to explore whether DC cytokine induction was affected by yeast viability. We observed no significant differences between the DC cytokine inducing properties of live, UV irradiated, and heat treated *S. boulardii*, and in addition, DC co-stimulatory functions were upregulated to an equal extent by live and reproductively unable yeast.

The remarkable consistency in DC cytokine inducing properties observed across very diverse yeast species and genera regardless of viability appears to support our hypothesis that interactions between yeasts and immune cells are likely to rely upon contact between (possibly conserved) yeast cell wall structures and DC surface receptors. Further, our findings suggest that this phenomenon holds true for a number of diverse yeast species beyond the widely studied *Saccharomyces* genus. In this context, it is interesting to note that a recent study have found yeast cell wall fractions, and particularly β-glucan fractions, prepared from either *S. cerevisiae* or *C. albicans*, able to modulate intestinal inflammation *in vivo* [40], potentially through interactions with the C-type lectin receptor Dectin-1 expressed by DCs [61], [62]. Thus, while our findings for nonviable yeasts beyond *S. cerevisiae* and *C. albicans* appear to extend their observation that yeast cell wall molecules are capable of interacting with immune cells, their findings support our hypothesis that yeast modulation of intestinal inflammation can take place without the need for live yeast cells.

In conclusion, the present study provides the first large-scale study of immune modulation by highly diverse yeasts described in the scientific literature. Our data clearly demonstrate high diversity in yeast induced cytokine secretion across a broad spectrum of yeasts, and by employing multivariate data analysis we reveal distinct clustering of yeasts inducing similar cytokine profiles in DCs, highlighting clear species distinction within specific yeast genera. The observed differences in induced DC cytokine profiles may indicate distinct modes of interaction between yeasts and human immune cells, and will aid in the selection of strains for further characterization and development toward potentially novel yeast probiotics. Additionally, we present data to support a hypothesis that the interaction between yeasts and human DCs does not solely depend on yeast viability, a concept which may suggest a need for further classifications beyond the current definition of a probiotic.

## Supporting Information

Figure S1 **Time-course of** ***S. boulardii*** **induced DC cytokine secretion supports a 20 h stimulation time.** Levels of IL-12, IL-10, IL-1β, IL-6, and TNFα secreted by human monocyte-derived DCs following incubation with *S. boulardii* (Ultra-Levure) at a yeast:DC ratio of 10∶1. Data are expressed as mean±SEM (n = 2).
(TIF)

Figure S2 **Modulation of DC co-stimulatory functions and chemokine receptor expression occurs independently of yeast metabolic function.** DC surface expression of CD80, CD86, CCR6, and CCR7 following 20 h stimulation with DC media containing 10% glycerol (unstimulated) or either live, UV irradiated, or heat killed *S. boulardii* (Ultra-Levure) at a yeast:DC ratio of 10∶1. Data are expressed as mean±SEM (n = 4). One-way ANOVA, Bonferroni’s multiple comparison post test, indicating significant differences between cytokine levels induced by live, UV treated, and heat killed yeast. ns, not significant; *, P<0.05; **, P<0.01; ***, P<0.001.
(TIF)
