# Supplementary figures and images for: Yeast Modulation of Human Dendritic Cell Cytokine Secretion: An In Vitro Study
Source: PLoS One. 2014 May 9;9(5):e96595. doi: 10.1371/journal.pone.0096595 (PMC4015989; doi:10.1371/journal.pone.0096595)

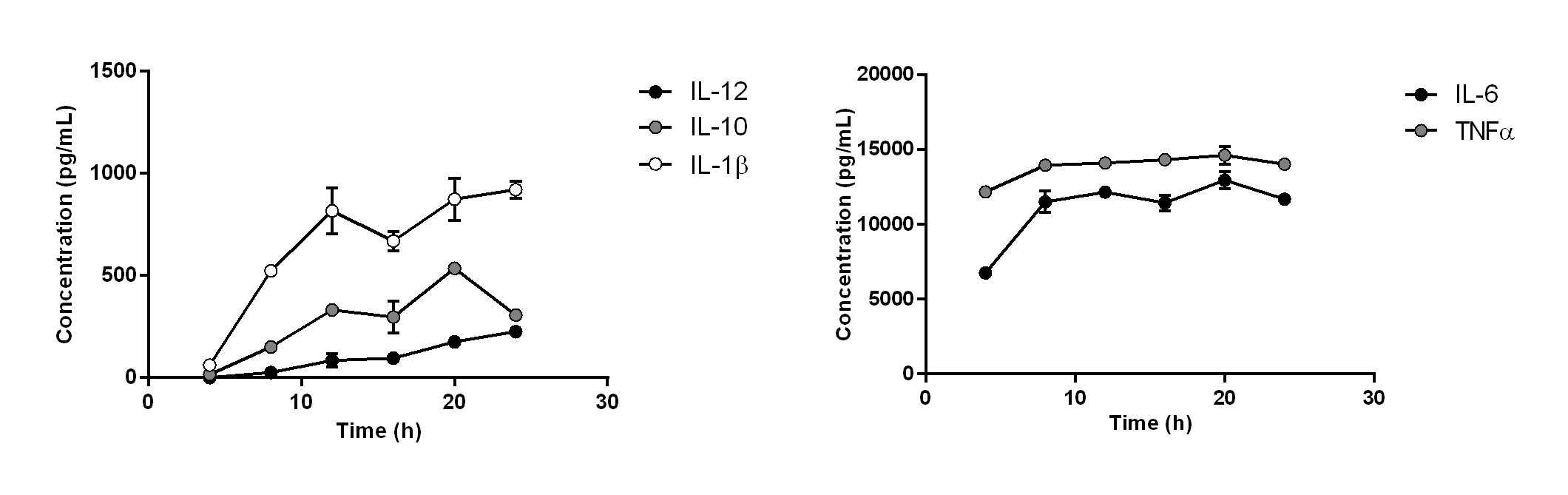

Supplement: Figure S1 — Time-course of S. boulardii induced DC cytokine secretion supports a 20 h stimulation time. Levels of IL-12, IL-10, IL-1β, IL-6, and TNFα secreted by human monocyte-derived DCs following incubation with S. boulardii (Ultra-Levure) at a yeast:DC ratio of 10∶1. Data are expressed as mean±SEM (n = 2). (TIF) [file pone.0096595.s001.tif]

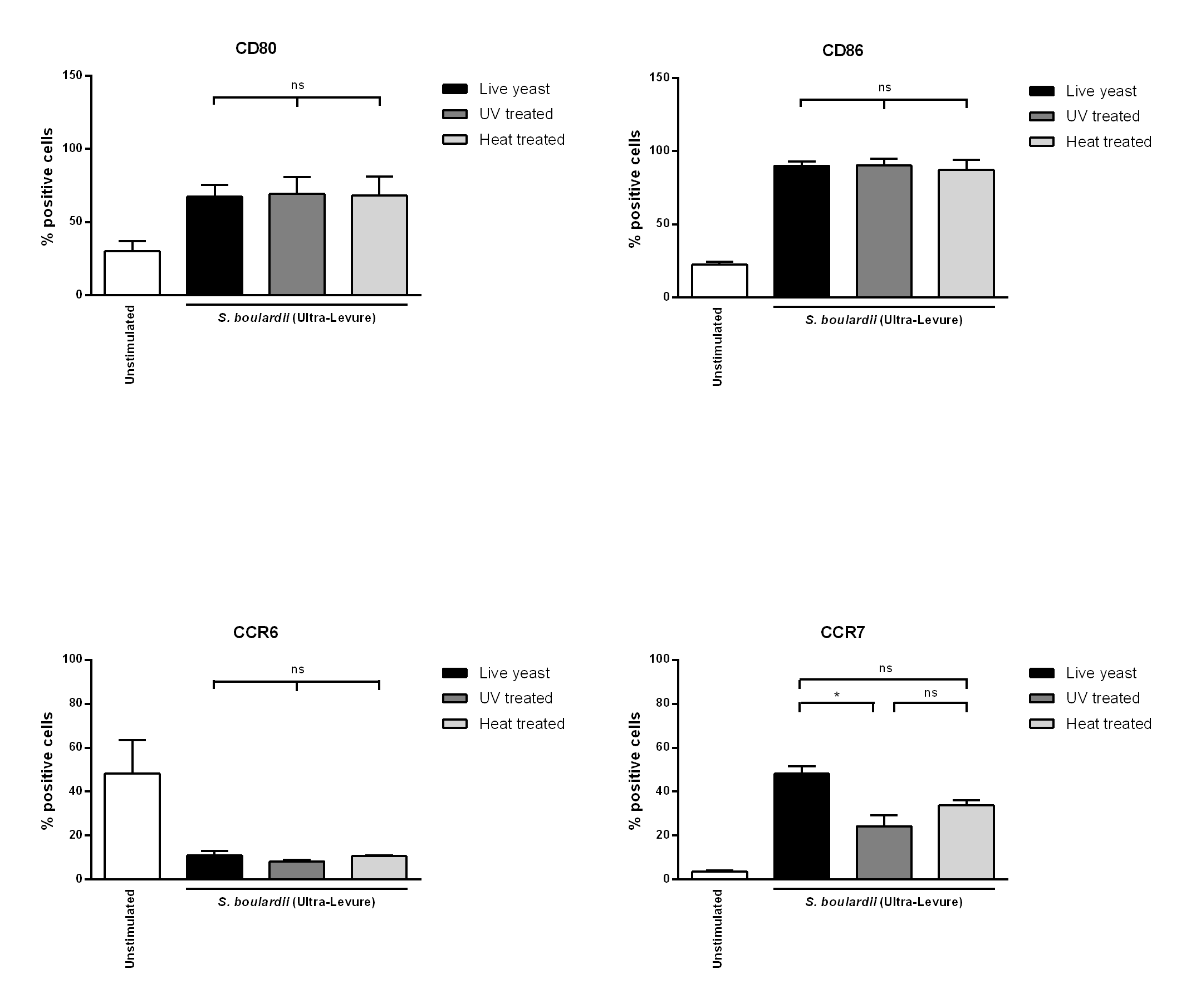

Supplement: Figure S2 — Modulation of DC co-stimulatory functions and chemokine receptor expression occurs independently of yeast metabolic function. DC surface expression of CD80, CD86, CCR6, and CCR7 following 20 h stimulation with DC media containing 10% glycerol (unstimulated) or either live, UV irradiated, or heat killed S. boulardii (Ultra-Levure) at a yeast:DC ratio of 10∶1. Data are expressed as mean±SEM (n = 4). One-way ANOVA, Bonferroni’s multiple comparison post test, indicating significant differences between cytokine levels induced by live, UV treated, and heat killed yeast. ns, not significant; *, P<0.05; **, P<0.01; ***, P<0.001. (TIF) [file pone.0096595.s002.tif]
